# Determinants of use of skilled birth attendant at delivery in Makueni, Kenya: a cross sectional study

**DOI:** 10.1186/s12884-015-0442-2

**Published:** 2015-02-03

**Authors:** Anne Gitimu, Christine Herr, Happiness Oruko, Evalin Karijo, Richard Gichuki, Peter Ofware, Alice Lakati, Josephat Nyagero

**Affiliations:** Amref Health Africa in Kenya, P.O. Box 30125-00100, Nairobi, Kenya

**Keywords:** Maternal Health, Skilled attendant, Delivery, Birth, Obstetric care, Kenya

## Abstract

**Background:**

Kenya has a maternal mortality ratio of 488 per 100,000 live births. Preventing maternal deaths depends significantly on the presence of a skilled birth attendant at delivery. Kenyan national statistics estimate that the proportion of births attended by a skilled health professional have remained below 50% for over a decade; currently at 44%, according to Kenya’s demographic health survey 2008/09 against the national target of 65%. This study examines the association of mother’s characteristics, access to reproductive health services, and the use of skilled birth attendants in Makueni County, Kenya.

**Methods:**

We carried out secondary data analysis of a cross sectional cluster survey that was conducted in August 2012. Interviews were conducted with 1,205 eligible female respondents (15-49 years), who had children less than five years (0-59 months) at the time of the study. Data was analysed using SPSS version 17. Multicollinearity of the independent variables was assessed. Chi-square tests were used and results that were statistically significant with p-values, p < 0.25 were further included into the multivariable logistic regression model. Adjusted odds ratio (AOR) and their 95% confidence intervals were (95%) calculated. P value less than 0.05 were considered significant.

**Results:**

Among the mothers who were interviewed, 40.3% (489) were delivered by a skilled birth attendant while 59.7% (723) were delivered by unskilled birth attendants. Mothers with tertiary/university education were more likely to use a skilled birth attendant during delivery, adjusted OR 8.657, 95% CI, (1.445- 51.853) compared to those with no education. A woman whose partner had secondary education was 2.9 times more likely to seek skilled delivery, adjusted odds ratio 2.913, 95% CI, (1.337- 6.348). Attending ANC was equally significant, adjusted OR 11.938, 95% CI, (4.086- 34.88). Living within a distance of 1- 5 kilometers from a facility increased the likelihood of skilled birth attendance, adjusted OR 95% CI, 1.594 (1.071- 2.371).

**Conclusions:**

The woman’s level of education, her partner’s level of education, attending ANC and living within 5kms from a health facility are associated with being assisted by skilled birth attendants. Health education and behaviour change communication strategies can be enhanced to increase demand for skilled delivery.

## Background

On a worldwide scale, significance progress has been made to date on the Millennium Development Goal (MDG) number 5, on improving maternal health [1] Globally, the maternal mortality ratio dropped by 45 per cent between 1990 and 2013, from 380 to 210 deaths per 100,000 live births. Despite progress in all world regions, the maternal mortality ratio in developing regions—230 maternal deaths per 100,000 live births in 2013—was fourteen times higher than that of developed regions, which recorded only —16 —maternal deaths per 100,000 live births in 2013 [2].

The majority of maternal deaths are entirely preventable. The presence of a skilled professional reduces the risk of complications becoming fatal and averts the risk of postpartum haemorrhage, which is viewed as the number one cause of maternal deaths globally [3,4].

Sub-Saharan Africa bears the largest global burden of maternal deaths at 56%, [5] yet it only accounts for 12% of the world’s population and 17% of all births in the world, [6]. According to the millennium development goals report (2014) by United Nations, most of the maternal deaths in 2013 took place in Sub-Saharan Africa (62%) and Southern Asia (24%) [2].

Kenya, like several countries in Africa, has alarming maternal health indicators, with a maternal mortality ratio of 488 per 100,000 live births, and its proportion of skilled birth attendance has remained below 50% for over a decade (45% in 1998, 42% in 2003, & 44% in 2008/9, according to Kenya’s Demographic and Health Survey (KDHS) [7]. This falls well below the country’s MDG 5 target of 90% by 2015. Kenya faces numerous challenges in curbing maternal mortalities, some of which include: insufficient skilled health personnel at health facilities, lack of basic and comprehensive emergency obstetrics care in many hospitals particularly in rural areas, poor infrastructure, inadequate budgetary allocations and low literacy levels among mothers as well as high poverty levels.

The maternal mortality ratio in Makueni County is 452 per 100,000 live births, while skilled delivery is at 36% [7]. Numerous reasons for low utilisation of skilled birth attendance in Kenya exist, including cost of service (perceived or real), the distance to the health facility, and quality of care [7].

In an effort to accelerate improvement of maternal health, Africa launched its road map for accelerating the attainment of the Millennium Development Goals related to Maternal and Newborn health in 2004, whose first objective was to provide skilled attendance during pregnancy, childbirth, and the postnatal period at all levels of the health care delivery system, using evidence-based standards of care [6].

Kenya also set out targets in its first Health Sector Strategic and Investment Plan 2012 – 2018, aimed at improving skilled delivery from 44% in 2012 (baseline) to 60% in 2015 (mid-term) and 65% in 2018 [8].

Even with adequately laid out plans to improve maternal health indicators through improving attendance of skilled delivery, many studies, largely focus on factors surrounding health systems, that affect utilisation of skilled birth attendants – user fee, physical access to health facilities including distance, and transport to the health facility [9]. However, the significant effect of socio-economic factors including level of education, and wealth index, on the decision by women to attend skilled delivery cannot be ruled out, as indicated in other studies [10,11].

In Makueni County, there is no existing data on contextual factors that lead to low uptake of skilled delivery in the County. This is a potential policy tool to facilitate the development of interventions aimed at improving uptake of skilled delivery in Makueni County, thereby contributing to the attainment of Kenya’s 90% target of MDG 5.

The objective of this study is to identify relationships between the mother’s characteristics, (Table 1) access to reproductive health services and the use of skilled birth attendants during delivery. The analysis will be used to make recommendations on improving maternal health in Kenya and increasing the access and utilisation of skilled birth attendants.

**Table 1:** **Characteristics of sample and factors associated with skilled attendance at birth**

**Mother**’**s characteristics Independent variable**				
	**N** **(%)**	**Outcome skilled (n) %**	**Statistical test p-value unskilled (n) %**	
Mother’s Education				
None	92 (8.2%)	18 (3.7%)	74 (11.7%)	(P < 0.001)
Primary	740 (66.1%)	291 (59.5%)	449 (71.3%)	
Secondary	230 (20.6%)	134 (27.4%)	96 (15.2%)	
Higher Education	57 (5.1%)	46 (9.4%)	11(1.71%)	
Partner’s Education				
None	73 (7.4%)	18 (4.3%)	55 (9.7%)	(P < 0.001)
Primary	559 (56.8%)	187 (45%)	372 (65.4%)	
Secondary	281 (28.5%)	162 (38.9%)	119 (20.9%)	
Higher Education	72 (7.3%)	49 (11.8%)	23 (4.0%)	
Religion				
Catholic	243 (21.8%)	102 (20.9%)	141 (22.5%)	P = 0.488
Protestant	839 (75.3%)	374 (76.8%)	465 (74.2%)	
Other	32 (2.9%)	11 (2.3%)	21 (3.3%)	(P > 0.25)
Employment Status				
Unemployed	717 (81.2%)	298 (76.2%)	419 (85.2%)	(P < 0.001)
Employed	70 (7.9%)	521 (13.3%)	18 (3.7%)	
Self-employed	70 (7.9%)	41 (10.5%)	55 (11.2%)	
Parity				
One	564 (46.5%)	282 (57.7%)	289 (39.0%)	(P < 0.001)
Two	548 (45.2%)	170 (34.8%)	378 (52.3%)	
Three or More	100 (8.3)%	37 (7.6%)	63 (8.71%)	
Access to Reproductive Health Services				
Independent Variable	N (%)	Outcome		Statistical Test P-Value
		Skilled n (%)	Unskilled n (%)	
ANC Attendance (Skilled)				
Yes	1,168 (96.4%)	483 (98.8%)	685 (94.7%)	P = 0.001
No	44 (3.6%)	6 (1.2%)	38 (5.3%)	(P < 0.25)
Number of ANC visits				
1-3 sessions	581 (49.8%)	220 (48%)	361 (51.0%)	P = 0.570
4 + sessions	585 (50.2%)	238 (52.0%)	347 (49.0%)	(P > 0.25)
Distance to Facility				
1-5 km	886 (73.1%)	370 (75.7%)	516 (71.4%)	P = 0.181
6 + km	326 (26.9%)	119 (24.3%)	207 (28.6%)	(P <0.25)

## Methods

### Sampling

The study was an analysis of secondary data for cross-sectional baseline survey conducted in August 2012 for an Amref Health Africa intervention project entitled *Mama na Mtoto wa Afrika* (Mother and Child of Africa). The outcomes of the *Mama na Mtoto wa Afrika* project focus on increasing the access and utilisation of maternal health services, and increasing the capacity of local health systems to provide quality services. The study was supervised by Amref Health Africa project team, and it aimed at establishing benchmarks for Maternal, Newborn and Child Health Services in Makueni County, Kenya.

The sampling frame consisted of the Population and Census Enumeration Areas (EAs) used in the 2009 Population and Housing Census in Kenya conducted by the Kenya National Bureau of Statistics. The primary sampling unit (PSU) referred to as a cluster in this survey was a village.

A two-stage sampling design was used. In the first stage, a random sample of villages was selected for each of the 5 district based on probability proportional to their population (PPP). The number of villages selected from each district was determined based on population weights from the 2009 Kenya Population and Housing Census which detailed the number of women and men per household in each locality. In the second stage, a minimum of 20 households were systematically selected from each village, (every 5^th^ household) in order to create a sample size of 1,181 households. Out of the targeted 1,181 women, a total of 1,205 women participated in the survey. The quantitative data collection was done through face to face interviews to eligible female respondents (15-49 years), who had children less than five years ago (0-59 months). If a household had two women who qualified for the study, then one was randomly selected.

### Study area

The household survey was conducted in Makueni County, which is located in the southern end of the Eastern Province in Kenya. The total population of Makueni is 884,527 with 11.8% living in urban areas. [12]. Makueni has a surface area of 8,009 km^2^ and a density of 110 people per kilometres-km^2^ [12]. Table 2 outlines the five districts, the total population and the number of households that were sampled.

**Table 2:** **District sampling**

**District**	**Total population of district**	**Sampled households**
Mbooni West	63,480	503
Mbooni East	77,271	281
Mukaa	83,776	187
Kathonzweni	101,240	53
Kilungu	122,500	181
**Total**	**448,267**	1,205

### Data collection

Data collection took place between August 13^th^ and 23^rd^, 2012. The survey questionnaire was adopted from the 2008/09 KDHS and was designed to permit the calculation of specific MNCH indicators. The survey tool was pre-tested before data collection began. Trained enumerators were responsible for collecting the data. The survey was in English; however, the enumerators were capable of translating questions into Kamba, the local language, when necessary.

### Data processing and analysis

The data was first entered into Census and Survey Processing System version 4.0 and then exported to Statistical Package for Social Sciences (SPSS) for further cleaning and analysis. Statistical analysis was based on the specific objectives of: identifying association between the mother’s characteristics and the use of skilled birth attendants during delivery and identifying associations between access to reproductive health services and the use of skilled birth attendants during delivery.

### Outcome variable

The primary outcome of interest is the use of a ‘skilled attendant’ at delivery, which “refers exclusively to people with midwifery skills (for example, doctors, midwives, and nurses) who have been trained to proficiency in the skills necessary to manage normal deliveries and diagnose, manage, or refer obstetric complications” [13]. Within the context of the household survey, traditional birth attendants “are excluded from the category of skilled attendant at delivery” [14]. This is aligned with Kenyan national policies, the World Health Organization and the United Nations Population Fund. The survey asked respondents ‘who assisted with the delivery of (name)?’ Within the analysis, doctors and nurse/midwives were considered as skilled attendants at delivery and traditional birth attendants, relatives, friends or any other individual was classified as unskilled.

### Predictor variables

The mother’s and partner’s levels of education were classified as; no formal education, primary, secondary or higher education. Mother’s religion was categorised as catholic, protestant or other. The employment status was classified as; unemployed (women with no employment), employed and self-employed. The number of births was grouped into 1, 2 and 3 or more. Use of ANC was divided into two; attending ANC sessions and not attending ANC sessions. The number of ANC visits was categorised into one-three visits and four or more ANC visits. The distance to a health facility was divided as 1-5 kilometres and above 6 kilometres.

### Statistical analyses

Data analysis was conducted using SPSS version 17.0. Complex Sample Analysis procedure was considered so as to adjust for sample weight, and multi stage sampling. An analysis plan was prepared using strata, cluster and sample weights. Univariate statistics was explored to determine the descriptive statistics.

For bivariate analysis, we used chi-square tests to measure the significance of relationships between the outcome variable and the predictor variables. The independent variables were mother’s level of education, partner’s level of education, employment status, number of births in the past five years prior to the study, ANC attendance, as well as distance to a health facility.

Multicollinearity of the independent variables was assessed. Results that were statistically significant with p-values, p < 0.25 were included into the multivariable logistic regression model. Adjusted odds ratio (AOR) and their 95% confidence intervals were calculated. A p value less than 0.05 were considered significant.

### Ethical considerations

Ethical approval for the secondary data analysis of the cross sectional cluster survey was provided by Amref Health Africa Ethics & Scientific Review Committee (ESRC). The ESRC has been appointed by the Kenya National Council of Science and Technology (NCST) as one of the Institutional Review Boards (IRBs) responsible for the ethical review process in Kenya.

## Results

A total of 1,212 women were included in the complex analysis out of which 489 (40.3%) were assisted by a skilled birth attendant at last delivery compared to 723 (59.7%) who were not.

### Characteristics of sample

Two thirds of the women, (66%) had primary education and 56.8% of the women’s partners had studied up to primary school as well. Slightly over three quarters, 81.2%, of the women were unemployed. Antenatal care was common, with 96.4% of women reporting that they attended skilled antenatal care sessions and 50.2% attending more than four ANC sessions. The distance to the nearest health facility, in kilometres, demonstrated that 73.1% women lived within one to five kilometres and 26.9% lived over six kilometres from a facility. The results for characteristics of sample for the outcome variable and the explanatory variables are show in Table 1.

### Correlation between independent variables

Multicollinearity of the independent variables was assessed. We considered a tolerance which was equal or less than 0.2 and VIF (Variance Inflation Factor) greater than 5, to depict multicollinearity.

The results from the collinearity statistics test established that most variables had a tolerance greater than 0.2 and the variance inflation factor was less than 5; hence there was no correlation among the independent variables. These variables were mother’s level of education and partner’s level of education, mother’s level of education and employment status, partner’s level of education and mother’s employment status.

### Factors associated with skilled attendance at birth

The mother’s level of education, partner’s level of education, employment status, number of births in 2007 or later, (as at the time of the study), ANC attendance, and distance to the nearest health facility were associated with skilled delivery, using chi-square tests. The p values were less than 0.25 (Table 1). Religion and number of ANC visits were not significant as their p values were greater than 0.25.

### Multivariable logistic regression

Using a backward elimination method multivariable logistic regression were developed (Table 3). Mothers who had a higher level of education (tertiary/university) were more likely to use a skilled birth attendant during delivery, (adjusted OR 8.657, 95% CI, 1.445- 51.853). Those with secondary level education were four times likely to use a skilled birth attendant (adjusted OR 4.662, 95% CI, 1.447- 15.024) while those with primary education were three times likely (adjusted OR 3.35, 95% CI, 1.168- 9.605) to use a skilled birth attendant during delivery as compared with those with no education at all.

**Table 3:** **Multivariable logistic regression by characteristics of sample and factors associated with skilled attendance at birth**

**Independent variable**	**Unadjusted odds ratio (95% CI)**	**P- value**	**Adjusted odds ratio (95% CI)**	**P- value**
Mothers’ Characteristics				
Mother’s Education				
None	Reference		Reference	
Primary	2.664 (1.366-5.197)	0.005	3.35 (1.168- 9.605)	0.026
Secondary	5.738 (2.743-12.005)	0.00	4.662 (1.447- 15.024)	0.012
Higher Education	17.192 (6.334-46.662)	0.00	8.657 (1.445- 51.853)	0.02
Partner’s Education				
None	Reference		Reference	
Primary	1.536 (0.876-2.693)	0.13	1.217 (0.658-2.25)	0.52
Secondary	4.160 (2.196-7.879)	0.00		0.009
			2.913 (1.337- 6.348)	
Higher Education	6.510 (2.742-15.455)	0.00	3.156 (1.087- 9.165)	0.035
Employment Status				
Self Employed	Reference		Reference	
Employed	3.875 (1.837-8.176)	0.001	2.117 (0.773- 5.799)	0.139
Unemployed	0.954 (0.57-1.597)	0.854	1.006 (0.505- 2.004)	0.987
Parity				
Three or More	Reference			
One	1.703 (0.799-3.631)	0.164	0.737 (0.267- 2.036)	0.545
Two	0.766 (0.353-1.662)	0.493	0.537 (0.203- 1.417)	0.201
Access to Reproductive Health Services				
ANC Attendance (Skilled)				
No	Reference			
Yes	4.466 (1.689-11.804)	0.003	11.938 (4.086- 34.88)	0.000
Distance to Health Facility in Kilometres				
1-5 km	Reference			
6 + km	0.575 (0.899-1.730)	0.181	1.594 (1.071- 2.371)	
	0.023			

The partner’s education level was equally significant in influencing the outcome variable. A woman whose partner had a higher education level (tertiary/college) was three times more likely to use a skilled birth attendant, (adjusted OR 3.156, 95% CI, 1.087- 9.165). A woman whose partner had secondary education was 2.9 times more likely to influence the outcome variable, (adjusted odds ratio 2.913, 95% CI, 1.337- 6.348) while one whose partner had studied up to primary school had a lower odds of influencing skilled birth attendance, (adjusted OR 1.217, 95% CI, 0.658-2.25) compared with a partner with no education.

ANC attendance similarly proved to be significant in influencing skilled birth attendance, (adjusted OR 11.938, 95% CI, 4.086- 34.88). The distance to a health facility greatly influences the use of skilled delivery. Living within a distance of five kilometers from a health facility was associated with a higher likelihood of skilled birth attendance, (adjusted OR 1.594, 95% CI, 1.071- 2.371) compared to living 6 Kilometres and above from a health facility.

## Discussion

The paper provides results on determinants of skilled attendance in Makueni County, in Kenya. Skilled birth attendant is important during pregnancy, at child birth as well as the post natal (after delivery) period. The study demonstrates that 43.7% of births within Makueni were attended by a skilled professional. These results are in line with the KDHS, whereby statistics for skilled attendants are 44% in Kenya and 43.1% in the Eastern Province [7]. The Makueni Multiple Indicator Cluster Survey from 2008 reports lower statistics for Makueni County with 36% of births attended by a skilled professional, one of the lowest percentages within the Eastern Province [15].

The multivariable logistic regression results relating to the mother’s level of education is similar to those in previous studies, that an increased level of education for a mother or her partner increases her chances of delivering with a skilled attendant [7,16,17]. The KDHS demonstrates that mothers with a higher education report higher rates of utilising a skilled attendant at birth: 72.5% for women who have completed secondary school, 48.9% for those who have completed primary school, compared to 28.5% among those who failed to complete primary schooling and 19.2% with no education [7]. The results from our analysis on the association between a woman’s education level and the use of a skilled birth attendant are consistent with a previous global study undertaken in Bangladesh [18].

The partner’s level of education is typically seen as important since it reflects the influence of the head of the household in making reproductive health decisions [19]. Partners who had higher education level (college/tertiary) as well as secondary education had higher likelihood of influencing skilled birth attendance. This is important as it reinforces the association between education and uptake of skilled delivery. Although the mother’s education level is significant in determining the use of a skilled birth attendant, the study suggests that the partner’s level of education is equally important. As a result, more emphasis is required in educating men specifically those with no education, as well as those with primary education, on the importance of skilled delivery services and its association with reduced maternal mortalities.

Receipt of ANC was high and this result is aligned with the Kenyan national statistics of 92% of women attending ANC [7]. The study reveals that ANC attendance influences skilled delivery since women are sensitised on the importance of skilled delivery during the ANC visits. When a woman decides to attend ANC, it also influences their decision whether to seek professional care during delivery.

While ANC attendance is high, there is a clear disparity with the number of women who follow through the continuum of care to skilled attended delivery. This disconnect is also evident in other previous studies from Kenya [7,20]. While it is recommended to begin ANC within the first trimester, only 15% of women in Kenya follow these guidelines [7]. The trend in delaying ANC has been seen within other research studies in Kenya [21]. It is plausible that the delay in ANC reflects the quality of care, the level of satisfaction with services provided and the behavioural attitudes towards the importance of ANC. Further qualitative research is needed in order to identify underlying reasons for women choosing not to attend timely ANC sessions during pregnancy.

Living six kilometres or more from a facility was associated with a reduced likelihood of skilled birth attendance. This can be attributed to the terrain in Makueni County; some sub counties are quite hilly and have impassable roads. The barrier effect of distance is stronger when combined with terrain and lack of transport such as ambulances to carry pregnant women to health facilities, in case they experience labour. The findings correspond to another study [17] which indicates a greater distance to a health facility significantly influences the mother’s choice of using a skilled birth attendant at birth.

### Limitations of the study

A key limitation of the research is the use of secondary data that had some missing values. We addressed this issue by using weighted analysis and complex sample analysis in SPSS to adjust for sample weight, and multi stage sampling.

Despite the study limitations, the results presented give an account of maternal and child health services within Makueni County, in Kenya.

## Conclusion

The study found out that there is a relationship between a woman’s education level, partner’s education level and the outcome of skilled delivery. Further attending ANC visits, and the distance to health facility were also strongly associated with utilisation of skilled delivery.

We found out that there are various barriers that influence the uptake of skilled delivery services in Makueni County. They include low education level for the mother and her partner (none or primary school level), not attending ANC sessions as well as a greater distance to the health facility (further than six kilometers).

Based on the findings, interventions in health related sectors such as health education and behaviour change communication need to be enhanced so as to increase demand for skilled delivery and ultimately improve maternal and child health; qualitative research including focus group discussions could provide insight into the gender and societal norms of the area as well other factors associated with skilled attendance at birth in Makueni. An assessment of the quality of facilities and available health care services would provide further insights relating to access of maternal, newborn and child health services.

## Abbreviations

ANC: Antenatal care
ESRC: Ethics and scientific review committee
IRB: Institutional review board
KDHS: Kenya demographic and health survey
MDG: Millennium development goal
MNCH: Maternal, newborn and child health
NCST: National council of science and technology
OR: Odds Ratio
SE: Standard Error
SPSS: Statistical package for social sciences
